# Periodontal ligament and alveolar bone remodeling during long orthodontic tooth movement analyzed by a novel user-independent 3D-methodology

**DOI:** 10.1038/s41598-023-47386-0

**Published:** 2023-11-14

**Authors:** Marta Rizk, Christian Niederau, Alexandru Florea, Fabian Kiessling, Agnieszka Morgenroth, Felix M. Mottaghy, Rebekka K. Schneider, Michael Wolf, Rogerio B. Craveiro

**Affiliations:** 1 Department of Orthodontics, University Hospital RWTH Aachen, Pauwelsstr. 30, 52074 Aachen, Germany; 2 Department of Nuclear Medicine, University Hospital RWTH Aachen, Aachen, Germany; 3 Department of Radiology and Nuclear Medicine, Academic Hospital Maastricht, Maastricht, The Netherlands; 4 School for Cardiovascular Diseases (CARIM), Maastricht University, Maastricht, The Netherlands; 5 Institute for Experimental Molecular Imaging, University Clinic Aachen, RWTH Aachen University, Aachen, Germany; 6 Institute of Cell and Tumor Biology, RWTH Aachen University, Aachen, Germany

**Keywords:** Experimental models of disease, Dentistry, Orthodontics

## Abstract

The structural process of bone and periodontal ligament (PDL) remodeling during long-term orthodontic tooth movement (OTM) has not been satisfactorily described yet. Although the mechanism of bone changes in the directly affected alveolar bone has been deeply investigated, detailed knowledge about specific mechanism of PDL remodeling and its interaction with alveolar bone during OTM is missing. This work aims to provide an accurate and user-independent analysis of the alveolar bone and PDL remodeling following a prolonged OTM treatment in mice. Orthodontic forces were applied using a Ni–Ti coil-spring in a split-mouth mice model. After 5 weeks both sides of maxillae were scanned by high-resolution micro-CT. Following a precise tooth movement estimation, an extensive 3D analysis of the alveolar bone adjacent to the first molar were performed to estimate the morphological and compositional parameters. Additionally, changes of PDL were characterized by using a novel 3D model approach. Bone loss and thinning, higher connectivity as well as lower bone mineral density were found in both studied regions. Also, a non-uniformly widened PDL with increased thickness was observed. The extended and novel methodology in this study provides a comprehensive insight about the alveolar bone and PDL remodeling process after a long-duration OTM.

## Introduction

Orthodontic theory and practice deal with bone remodeling and require a thorough understanding of bone biology, particularly the relationship between mechanical stress and various cell types in bone^1^. Although in the clinical praxis the magnitude, direction, and duration of the applied force can be relatively well regulated, due to limitations of examination in patients, we still do not have enough knowledge about the changes in alveolar bone and periodontal ligament microstructures. From this reason, animal experiments are essential to elucidate interaction between periodontal ligament and alveolar bone during orthodontic tooth movement (OTM).

During OTM, the teeth are loaded with a specific mechanical force through orthodontic intra- and extraoral appliances in a coordinated complex process. As such, OTM supports the adaptation of different cell populations into the tissue microenvironment within the periodontal ligament (PDL) and alveolar bone, which leads to particular remodeling. This mechanical force triggers stress and strain distribution in the PDL, causing local hypoxia and fluid flow, initiating a sterile inflammation, which in the end enables bone remodeling. Bone resorption occurs on the compression side along with resorption of the alveolar bone and degradation of the periodontal ligament, while new bone is formed on the tension side with stretch of the periodontal ligament inducing bone apposition and alignment of the Sharpey fibers^2–4^.

Although the PDL plays a unique and dominant role in the regulation of bone remodeling during OTM^5–8^, there are no studies comparatively assessing the crosstalk between PDL and bone in long-time periodontal tissue remodeling. A detailed characterization of PDL regeneration and remodeling in its complexity in later phases is required to understand the complex biological mechanism of OTM.

Based on our previous study presenting an approach to follow the complexity and dynamics of OTM over a long time with non-invasive in vivo monitoring^2^, the aim of this study was to describe the changes of alveolar bone and PDL structures in a long duration OTM mice model by an accurate and user-independent methodology.

Micro-computer tomography (micro-CT) offers a non-destructive method of detailed anatomical assessment and is commonly used for bone remodeling assessment under OTM treatment in mice or rats^9,10^. So far, the methodologies used for bone and PDL microstructure evaluation in the literature vary in several aspects, which worsens the comparability between studies^11^. Still, the method of data analyses is crucial to the validity of the study. Various sizes and shapes of volumes of interest (VOI) for the morphometric analysis result in non-comparable, often user-dependent data. Typically, in a preclinical setup, bone remodeling is investigated in the alveolar socket of the OTM treated first molar (M1). Due to the intermittent connection and interaction between all bone areas and PDL in the orthodontically treated periodontium, this tissue may also be affected in the surrounding region of the treated tooth. Still, literature provides insufficient knowledge about the cellular and morphological response in such surrounding areas. Additionally, bone remodeling differences in dependence of the stress distribution in the orthodontically treated periodontium have been shown previously^12–15^. A prevalent compression force is known to lead to bone resorption while bone growth occurs predominantly on the tension side^4,12,14^. However, the exact definition of the location and processes of bone reformation in tension or compression side, respectively, remains insufficient^16,17^. We therefore plan to present an innovative insight about the various bone remodeling extent in diverse regions—alveolar socket of the treated M1 where a combination of both tension/compression regions is expected; alveolar bone between the first and second molar with presumably a tension region in dominance; and in periodontal ligament of M1 where both forces play their role.

During all OTM phases, bone and PDL undergo structural remodeling, characterized by changes in their porosity, mineralization, size, form, and pore distribution. These properties are well described through several parameters estimated via micro-CT. An increase in porosity can be defined by decreasing bone volume to total volume ratio (BV/TV). In a process of bone loss or an initial phase of bone growth, the trabecular bone typically reforms to thinner bone structures and larger space between these trabeculae, well defined by trabecular thickness (Tr.Th) and separation (Tr.Sep), respectively^13,18^. A higher density of pores often leads to extra connections between trabeculae, increasing the intra trabecular connectivity^19^. Still, the outcomes variability found in literature indicates the importance of studied parameters, such as duration of OTM, applied mechanical force, and evaluated VOI position and size^16,18,20,21^. Reduced bone mineral density (BMD) and BV/TV combined with a later thinning of trabeculae were found in several studies^13,20–26^ while other studies detected no changes in these parameters^16,18^. Also, the variability of the studied OTM time-points was shown to result in modified bone and PDL characteristics, pointing out their dynamic and often non-linear nature of bone and PDL remodeling^16,27–29^. For a proper and extensive understanding of bone and PDL remodeling and their possible interactions during OTM, it is of paramount importance that these oral processes would be carefully and extensively investigated in various regions of periodontium, and—particularly—at longer OTM durations.

The compression or extension within the PDL has been mostly demonstrated as a change of PDL thickness in the direction of the orthodontic force^12,25,29^. Such conclusions are typically estimated from individually selected 2D transverse sections of the treated molar^25,29,30^. Technical difficulties in the exact determination of the OTM force direction in 3D data may result in erroneous PDL thickness estimation. Discrepancies in quantitatively calculated PDL thickness may also be affected by individual selection of studied section along the roots. Also, a non-uniform stress distribution inside the PDL volume during various OTM forces, which has been previously shown by finite elemental analyses^31^, indicates the outcome dependence on the studied PDL-region. Overall, only a few studies applied more complex 3D methods to study the PDL changes^32–34^. All these aspects point out the necessity to establish a precise and reproducible methodology for studying the PDL changes inside the complete PDL volume during orthodontic stimuli. To gain a profound insight about the periodontal ligament during orthodontic treatment, a novel 3D approach for the characterization of the complete PDL space and its changes was implemented in this work.

Our study offers a unique characterization of alveolar bone remodeling during OTM in mice over a long time period of 5 weeks and provides a better insight into the later phases of alveolar bone and PDL remodeling. The macroscopic modifications of the PDL as a complete region were precisely studied as well. The focus of our study was the accurate and user-independent characterization of several periodontium areas around the orthodontically treated upper first molar tooth and on differences in the tissue remodeling processes affected by compression or tension forces in these regions. Finally, this study offers an important improvement step for the methodology applied for volumetric micro-CT studies of the periodontium during orthodontic therapy.

## Material and methods

### Ethical statements

The animal study protocol was approved by the competent authority and performed in compliance with the German Animal Protection Act (approval ID: 81-02.04 20190.A190, committee of North Rhine Westphalia, Germany). All experimental methods were performed in accordance with ARRIVE guidelines. All experiments were carried out following relevant guidelines and regulations.

### Experimental animals

For this study, mice with long term OTM treatment (5 weeks) were investigated^2^. Briefly, three male C57BL/6JRj wild-type mice (WT) (age: 10–11 weeks, Janvier Labs, Le Genest-Saint-Isle, France) were used for the OTM model. The first upper left molar was set under a constant force of 0.25N by a nickel-titan coil spring for anterior movement. The contralateral unstimulated side served as a control (split-mouth model). After the completion of 5 weeks of orthodontic treatment and non-invasive scans, all mice were euthanized. Then, the maxilla was removed and fixed in 3.7% paraformaldehyde solution for at least 24 h. Following, the samples were stored in 75% ethanol for micro-CT scan.

### Micro-CT scans

Maxillae (n = 3) were scanned in Skyscan 1272 (Bruker MicroCT, Belgium) at 60 kV, 166 µA, using the 424 ms integration time resulting in the isometric voxel size of 3 µm. Data were reconstructed by NRecon software (Bruker MicroCT, Belgium) and evaluated for the microstructural and PDL parameters using CTan software (Bruker MicroCT, Belgium) in a transverse view. To obtain 3D images, CTvox (Bruker MicroCT, Belgium) was used for 3D rendering.

### 3D analyses

**Figure 1:**
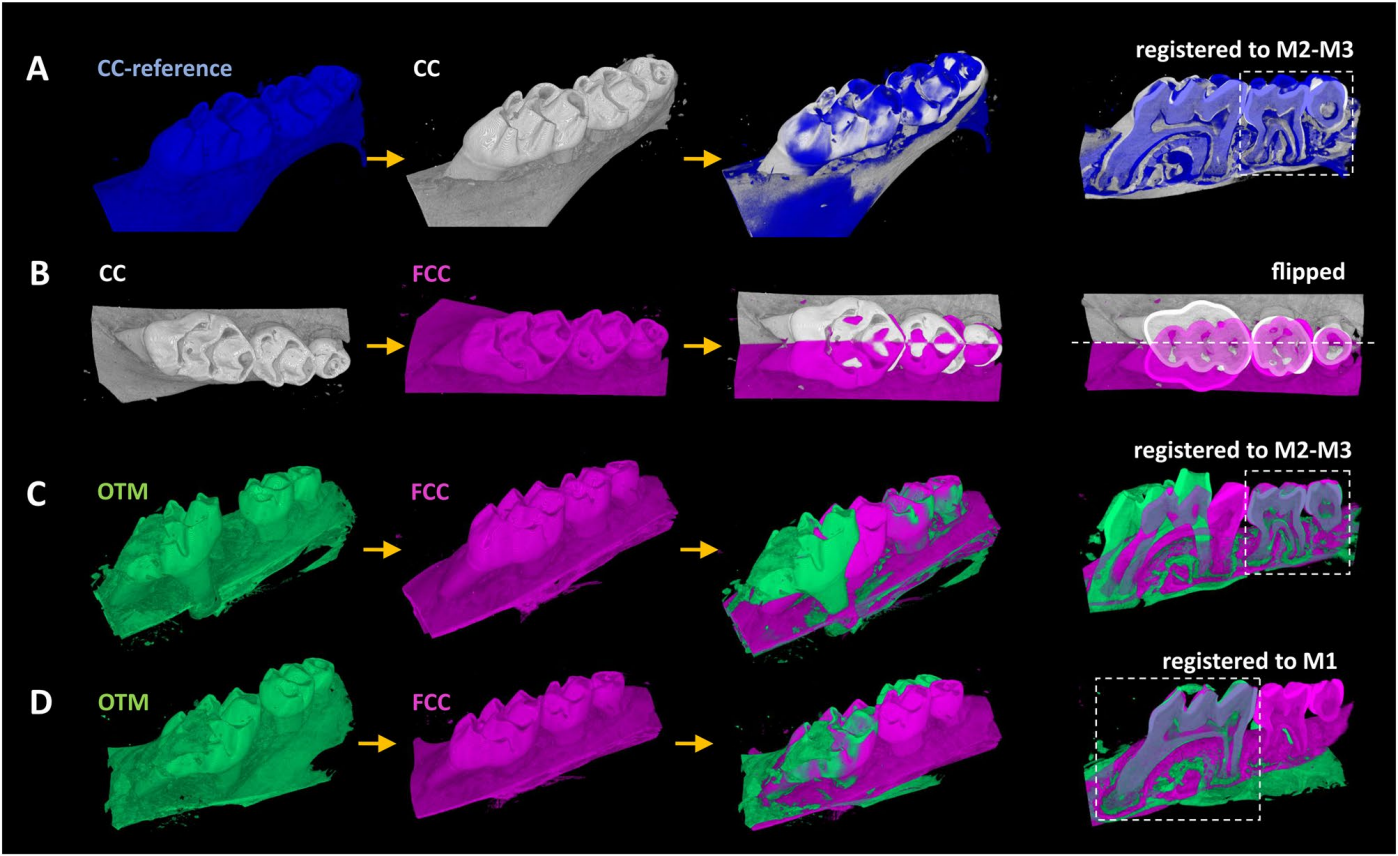
Schematic representation of the algorithms for the estimation of tooth movement and setup for the reproducible and identical localization of the studied VOIs. (**A**) Firstly, the CC sides (white) were registered in 3D to the CC-reference (blue) according to M2–M3 region. (**B**) Following, these CC sides (white) were geometrically flipped to FCC (rose) over the depicted sagittal plane in transverse view. (**C**) OTM sides (green) were registered to the FCC (rose) according to the M2–M3 region. (**D**) The tooth movement was defined from the further 3D-registration of OTM to FCC according to M1 region, as the difference between the final OTM position and its position in (**C**).

The upper jaw area of the 1st (M1) to 3rd (M3) molar were studied. The changes of OTM (right) side with spring coil were compared to contralateral side as a control (CC) (Fig. 1A).

### Tooth movement estimation

One control side (CC) scan was chosen as a reference for the 3D registration (DataViewer, Bruker MicroCT, Belgium) of the scans to ensure the same jaw orientation for all scans. Following, the rest two CCs were registered to this CC-reference according to the area of the second and the third molar (Fig. 1). Afterwards, all CC sides were geometrically flipped over a sagittal plane in transverse view to mimic the opposite (OTM) side using the geometrical transformation algorithm (CTan, Bruker MicroCT,Belgium). Each OTM side was then registered to its flipped CC (FCC) side according to the M2–M3 region. Afterwards, the M1 movement (translational and rotational) was estimated by the additional 3D registration inside the M1 region from the initial and final position matrix of the 3D data (DataViewer, Bruker MicroCT,Belgium). (Fig. 1).

### Morphometric analysis

To ensure the reproducibility of the morphological analysis through the automatic localization of the analyzed region (VOI), all data were initially registered in 3D (DataViewer, Bruker MicroCT, Belgium) to reach the most possible identical orientation. The OTM data were registered to their contralateral flipped control side (FCC) according to the 1st molar region for the analysis of alveolar bone between the mesiobuccal and distobuccal root of M1, presumably with the combination of tension and compression subregions (Fig. 1D). In that way, a single VOI could be chosen for all data while its reproducible and exact position was ensured. The registered data were then analyzed in CTan (Bruker MicroCT, Belgium) for bone mineral density (BMD) and microstructural parameters, inside the cylindrical volume of interest (VOI:M1), to obtain the following characteristics—bone volume/total volume (BV/TV); trabecular thickness (Tb.Th); trabecular separation (Tb.Sep); intratrabecular connectivity, defined as a number of independent connections within a complex bone structure (connectivity). BMD was normalized to the average BMD of control group as relative BMD. The VOI:M1 consists of a cylindrical cut with an area of 1.0 mm^2^ and a height of 0.9 mm (Fig. 3A). The structural parameters were estimated after virtual removal of tooth and soft tissue (pulpa and PDL) from VOI:M1 using a combination of thresholding and seed-growing algorithm (ROI-Shrink/Fill out option); and an algorithm for closing of the broken pores on the PDL/cementum/dentin border. A constant threshold for delineation between hard and soft tissue or fluids for all the scans was chosen for more precise comparison. The same parameters (BV/TV; Tr.Th; Tr.Sep and connectivity) were estimated for the bone area between the 1st and the 2nd molar tooth—VOI:M1-2 for the study of the bone under prevalently tension force (Fig. 6A). For the definition of the VOI:M1-2, the data registered to the M2–M3 were considered, as shown in Fig. 1C. The cylindrically shaped volume of interest with the height of 0.63 mm and a circular cut area of 1.05 mm^2^ included the alveolar bone between both molars and partially the bone around the roots of M1. A slightly smaller VOI was chosen for M1-2 to exclude the bone region under the roots.

### Periodontal ligament

For the characterization of PDL, a cylindrical VOI similar to VOI-1 but with larger cross-section to cover the complete space of the 1st molar and the surrounding PDL was considered as the volume of interest.

### Statistical analysis

To estimate the minimal sample size, power analysis was performed using G*Power software (G*Power 3.1.9.2, F.Faul, University Kiel, Germany). Since no OTM analyses after 5 weeks of treatment has been published yet, the power analyses with a significance level of 5% and power of 80% were done based on the results of BV/TV from Kako et al.^35^ for only 3 weeks of OTM (50.7 ± 3.1 for control group; 30.5 ± 1.7 for OTM group) resulting in the requirement of minimal sample size of 2 only. Based on this requirement, n = 3 was used in this study.

The statistical analysis was performed using GraphPad Prism (version 9.4.1) due to the small sample size. The inter-group comparisons were estimated based on independent unpaired two-tailed Student’s t-tests (α = 5). All data are presented as mean ± standard deviation (SD).

## Results

### Long OTM results in a complex tooth movement in mice

**Figure 2:**
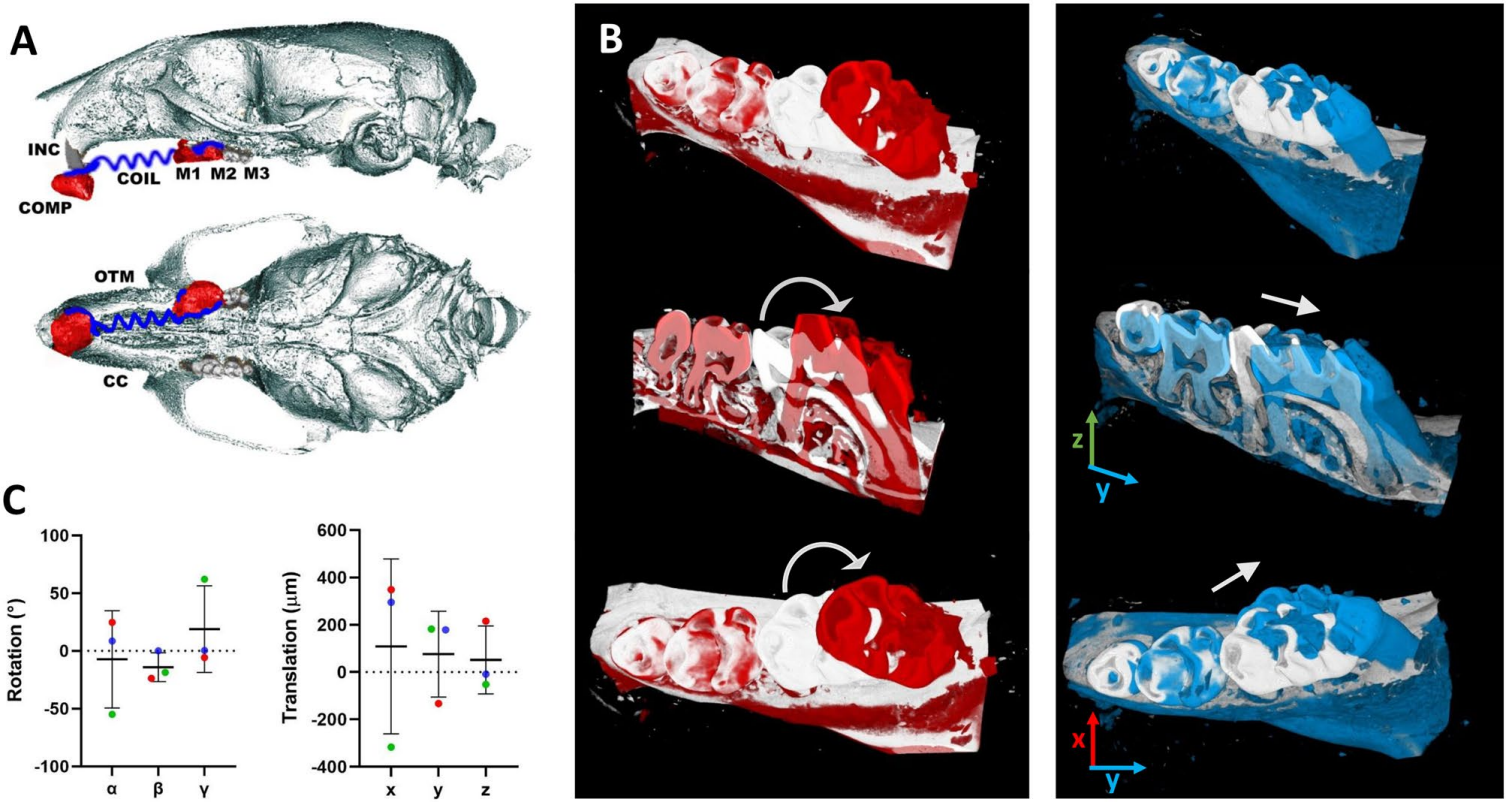
Tooth movement during a long OTM treatment. (**A**) Schematic representation based on micro-computed tomography scans showing 3-dimensional sagittal and occlusal views of an orthodontic appliance used in mouse. A depiction of the Ni–Ti coil (COIL) attached from upper incisor (INC) with composite (COMP) and to the first molar (M1). The region of the first to the third molar (M1, M2, M3) was used for analysis. The lower view of the maxilla shows that the left side served as a control (CC) and the right side was orthodontically treated (OTM). (**B**) A complexity of the tooth movement during a long-term OTM is shown on two examples. A strong rotational factor is visible on the red-colored sample after 5 weeks OTM in comparison to the white-colored flipped CC. A prevalent translational movement was found in one sample—blue-colored OTM in comparison to the white-colored flipped CC. The axes are demonstrated by the colored arrows and were defined as: x (red)—from buccal to palatal; y (blue)—from distal to mesial; z (green)—from vertical (from apical to occlusal). The translational movement and rotation around the x and z axes are symbolized by the white arrows. (**C**) The non-conformity of the translational movement after 5 weeks of OTM is depicted in the large deviations of the average translation in all three axes. The complexity of the tooth relocation is confirmed by the non-zero rotation angle with strong deviations, mainly around the x and z axis (γ and α, resp.).

The process of 3D overlapping of a geometrically flipped CC and an OTM group before the tooth movement estimation and setting up the VOI for the morphological calculations ensured a precise comparison. The complete tooth movement after 5 weeks of treatment was found to be complex and individual. One sample showed prevalently translational movement (blue-colored images in Fig. 2B), the other two maxillae showed a strong rotational aspect additional to the translation. Also, the rotation direction rotation varied strongly (Fig. 2C). High variations in translational and rotational movements indicate a rather complex nature of the tooth movement during such long orthodontic treatment. (Fig. 2).

### Alveolar bone underwent significant morphological changes

**Figure 3:**
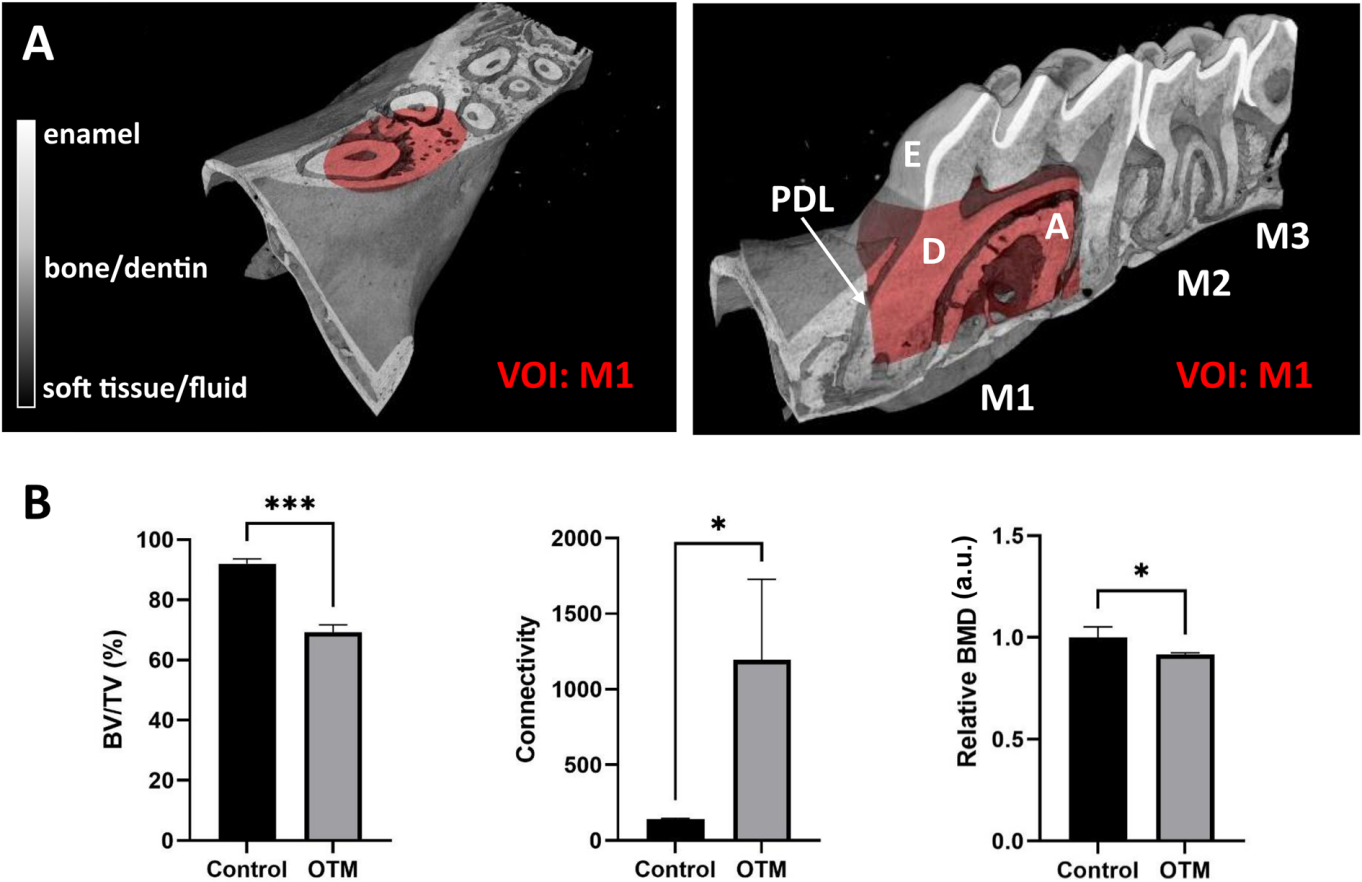
Microstructures morphometry of the alveolar bone during OTM. (**A**) Inside the volume of interest (VOI:M1) for microstructural analysis of the alveolar bone in the 1st molar tooth region—1st, 2nd and 3rd molar (M1, M2, M3, resp.); enamel (E), dentin (D), alveolar bone (A), periodontal ligament (PDL). (**B**) Data shows that bone/total volume (BV/TV) and BMD normalized to the BMD of control group (relative BMD) were significantly lowered after 5 weeks of the treatment while the intratrabecular connectivity strongly increased (B) (a.u. = arbitrary units). Inside the volume of interest (VOI:M1) for microstructural analysis of the alveolar bone in the 1st molar tooth region—1st, 2nd and 3rd molar (M1, M2, M3, resp.); enamel (E), dentin (D), alveolar bone (A), periodontal ligament (PDL). (**A**) data shows that bone/total volume (BV/TV) and BMD normalized to the BMD of control group (relative BMD) were significantly lowered after 5 weeks of the treatment while the intratrabecular connectivity strongly increased (**B**) (a.u. = arbitrary units), *p < 0.05, **p < 0.01, ***p < 0.001.

The VOI:M1 revealed highly significant changes in the BV/TV ratio. Also, relative BMD and connectivity resulted in significantly changed values after 5 weeks of OTM (Fig. 3B). The changes in the bone mineral density are less pronounced, but still follow the expected trend of bone mineral loss during the induced bone remodeling and are parallel with the BV/TV changes. Bone connectivity showed to be a sensitive parameter to explore bone morphometric changes, which, on the other side, led to strong deviations. Still, the higher porosity accompanied with a strong increase in connectivity indicates that after 5 weeks of OTM the appearance of new pores and widening of the existing neighboring ones were extended enough to cause a more complex trabecular structure. (Fig. 3).

**Figure 4:**
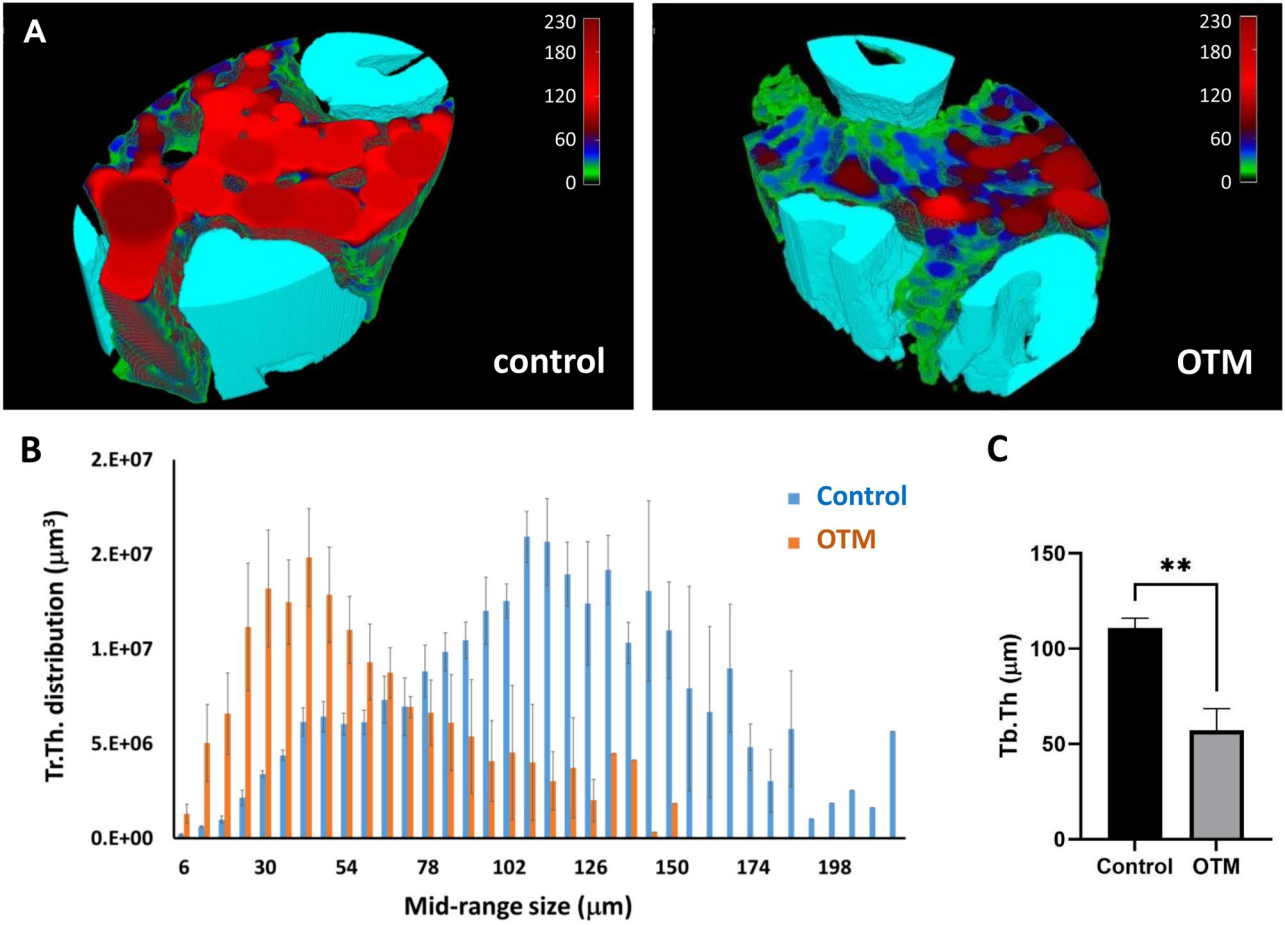
Changes of bone loss and thinning of the alveolar bone during OTM. (**A**) 3D rendering of the bone structure inside VOI:M1 with color-mapped trabecular thickness representation (Tr.Th) shows clear differences in the density of the thicker trabeculae for both groups. The color-mapped scale presents the distribution of Tr.Th in µm. (**B**) An average volume distribution of the trabecular thickness in VOI:M1 after 5 weeks of OTM covers mainly smaller Tb.Th values when compared to the non-treated side (control)—thinning of the bone structure. (**C**) The average trabecular thickness (Tr.Th) of the alveolar bone inside M1 region was also strongly reduced, **p < 0.01.

Bone loss during OTM was found to appear parallel to the thinning of alveolar bone inside the M1 root system (Fig. 4A). Visibly less trabeculae with the Tr.Th over 45 µm (red color-mapped regions) were found in the OTM sample. This alveolar bone consists of the trabeculae with the average thickness of (57 ± 11) µm. Contrarily to that, the control trabeculae resulted in the significantly higher Tr.Th of (111 ± 5) µm (Fig. 4C). The graph of the average Tb.Th volume distribution shows the shift from a relatively wide thickness distribution in control to the predominantly thinner sizes due to OTM treatment (Fig. 4B).

**Figure 5:**
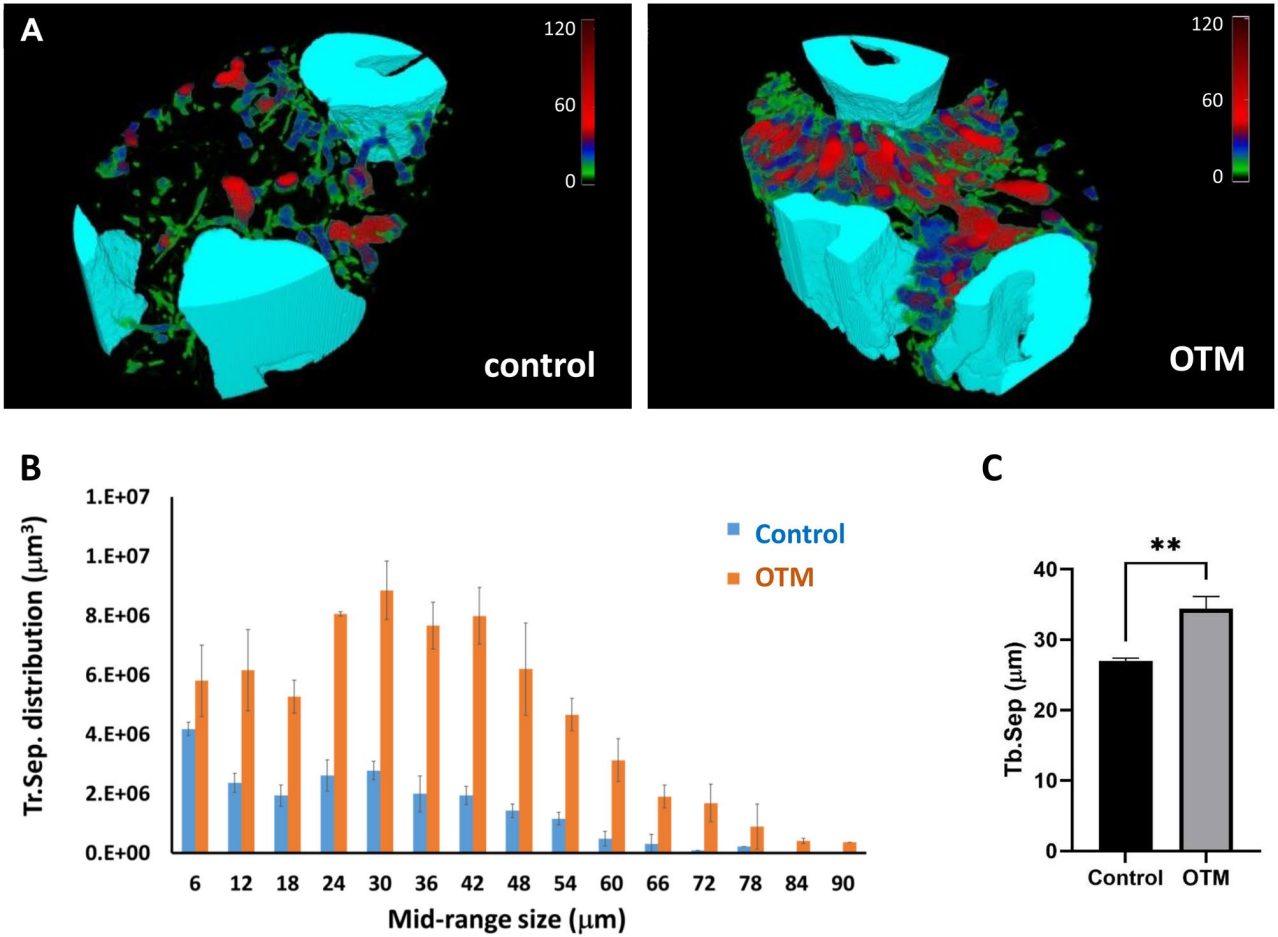
Changes in trabecular separation during OTM. (**A**) Color-mapped presentation of the trabecular separation (Tr.Sep) distribution inside VOI:M1 indicates a strong increase in the density and dimension of the pores after 5 weeks of OTM. The color-mapped scale shows the Tr.Sep distribution in µm. (**B**) The average volume distribution of Tr.Sep in VOI:M1 were confirmed with visibly lower small pores amount, and more middle-sized pores appeared in the treated side comparing to the control. (**C**) The average Tr.Sep was also found to be significantly higher in the OTM group, **p < 0.01.

Alongside the higher porosity and bone thinning after 5 weeks of OTM (Figs. 3 and 4), the expansion of the Tr.Sep in volume (Fig. 5B) reveals that some bone regions are replaced with pores. A higher density and larger dimensions of pores compared to the control can be seen too (Fig. 5A). The Tb.Sep volume distribution exhibits a slight shift of its center to the region with higher pore sizes. The overall volume was larger in the OTM group, which confirms the findings of decreased BV/TV shown in Fig. 3B. in the control. The smallest pores were defined by a diameter of 6 µm surpass while larger pores prevalence of 24–42 µm can be observed in the OTM group. These findings have been confirmed by the statistical outcome of significantly increased Tr.Sep within the OTM group (Fig. 5C).

The thinning of the trabeculae is well visible on the analyte 3D images (Fig. 4A) and in good agreement with the increased density and size of the pores that replaced the missing trabeculae (Fig. 5A). Thicker trabecular structures with a thickness of over 150 µm practically disappeared from the VOI:M1 after 5 weeks of OTM (Fig. 4B) while most of the trabecula reduced its thickness to the values around 40 µm only. Also, the size distribution of Tr.Th became visibly narrower following OTM. In the region between M1 and M2, this thinning was less evident. The variability of the pore sizes remained the same while significantly more pores appeared in the VOI:M1 (Fig. 5B). At the same time, the ‘center’ of distribution was shifted to the higher Tr.Sep values. The distribution shown in Fig. 5B demonstrates the importance of such characterization towards these parameters. That is to say, even with a minor change in the size distribution of Tr.Sep and thus of the average Tr.Sep, firm morphological changes may take place. (Fig. 5).

### Similar structural changes found in both studied areas—M1 and between M1 and M2

A missing convention about the morphometric evaluation of bone remodeling under OTM treatment raises the question, which alveolar bone of OTM side is predominantly modified by the bone resorption and where the bone growth prevails. Additionally, it is unclear which region undergoes the strongest structural changes. Therefore, beside the typically studied M1 region, the alveolar bone between the 1st and the 2nd molar (VOI:M1-2) were investigated in our study.

**Figure 6:**
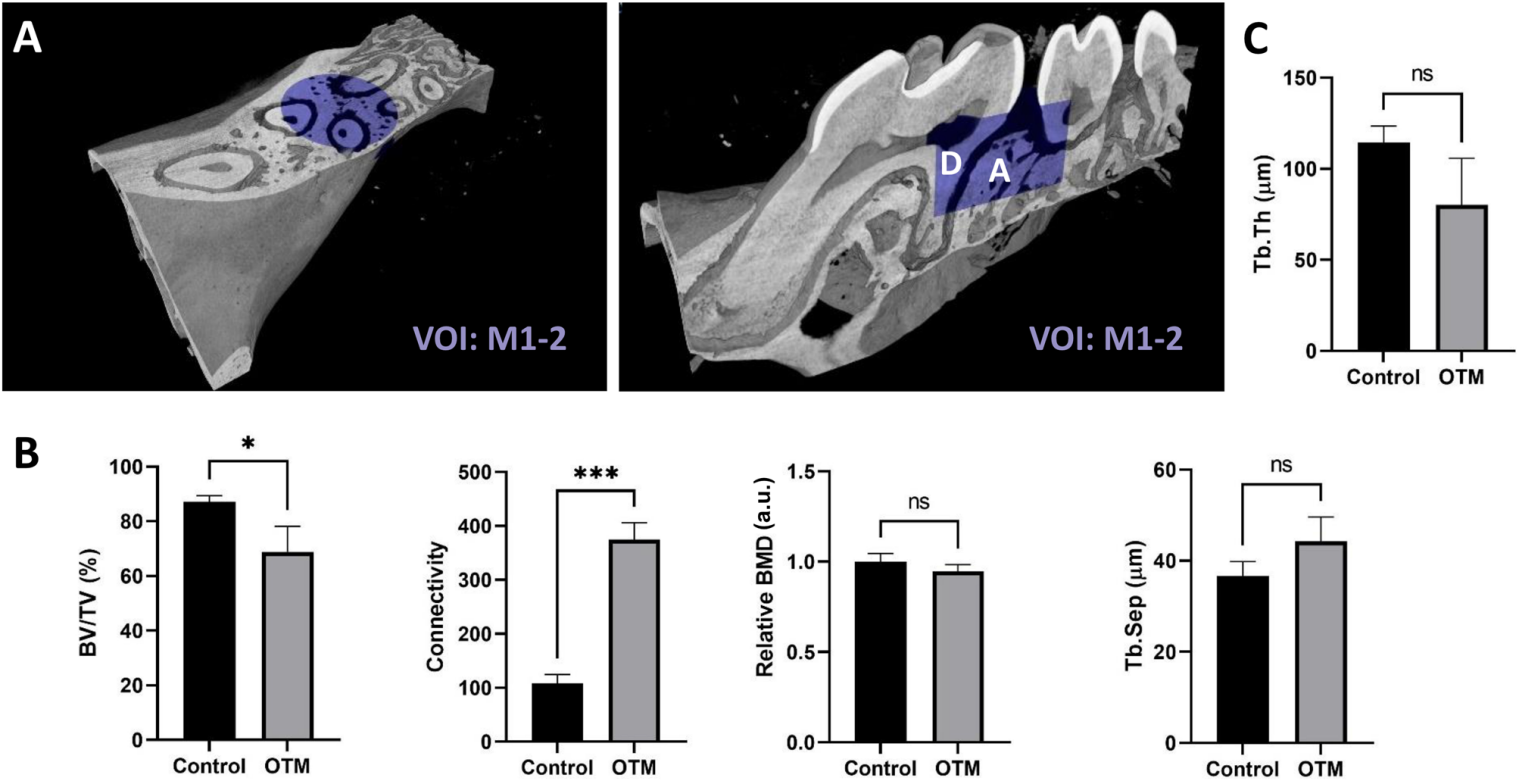
Structural changes in areas between M1 and M2. (**A**) The alveolar bone between the first and the second molar was evaluated inside the VOI:M1-2—dentin (D), alveolar bone (A). (**B–C**) Morphometric analyses showed less pronounced but comparable changes in all studied parameters as in VOI:M1, *p < 0.05, **p < 0.01, ***p < 0.001**.**

Based on the similarity showed by the trends in all evaluated parameters to the results from the VOI:M1 region (Fig. 6B), seemingly similar bone remodeling changes proceeded in both regions during the 5 weeks of OTM. Increased porosity, Tr.Sep and connectivity together with the decreased Tr.Th confirmed the bone resorption process. Still, based on the weaker statistical differences, these changes may be less pronounced in this volume of interest in comparison with VOI:M1 (Figs. 3, 4, 5 and 6).

### Periodontal ligament complex expansion after OTM

**Figure 7:**
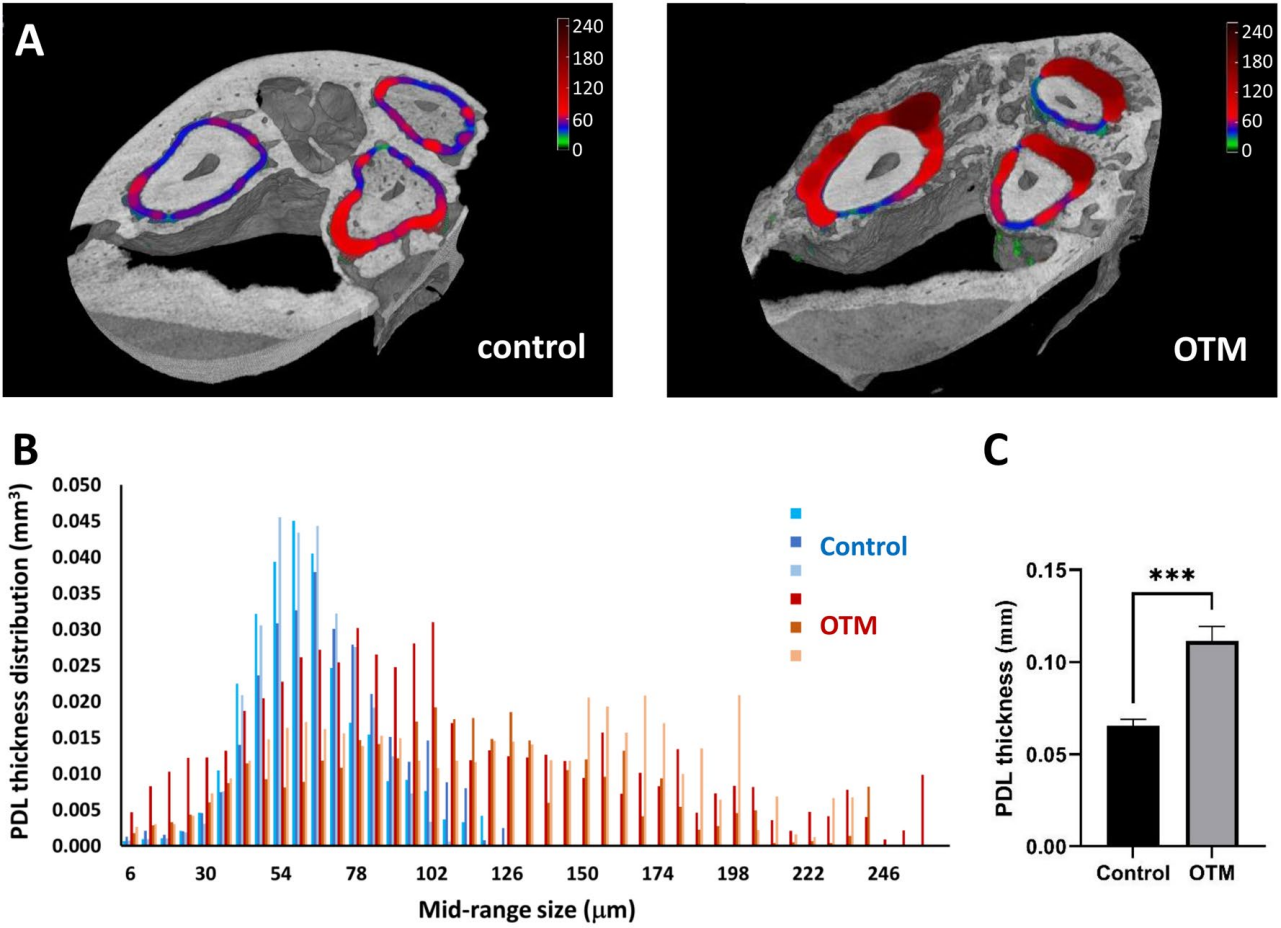
Periodontal ligament complex expansion following OTM. (**A**) Widening of the PDL layer as seen on the PDL thickness distribution depicted on the color-mapped 3D rendering images. The color-mapped scale bar shows the PDL thickness in µm. (**B**) The volumetric distribution of PDL thickness where a relatively thin distribution of the control group spreads towards the higher values of PDL thickness. (**C**) t-test confirmed the significant increase in PDL thickness, ***p < 0.001.

Due to the inherent connection between bone and PDL inside of periodontium, the periodontal ligament was evaluated for possible remodeling too. PDL volume and thickness were increased in a similar manner following OTM treatment (Fig. 7C), while only the PDL thickness changes were found to be significant (*p* = 0.0007). This thickening of PDL, together with its deformation, can be observed in 3D rendering with a color-mapped depiction of PDL thickness (Fig. 7A). The volumetric distribution of PDL thickness (Fig. 7B) confirms the malformation by a strong distribution widening in all OTM samples in comparison to the relatively narrow distribution located around thinner PDL thickness values in control group (Fig. 7).

## Discussion

Long-term orthodontic treatment in mice or rats over 3 or more weeks is scarce due to the challenging experimental setup^2,16^. We have successfully proceeded the longest studied OTM experiment in mice so far^2^ and could profoundly investigate the bone and PDL changes after this extended mechanical stimulus, with clear alterations in tooth position, alveolar bone, and PDL morphology. The differences between the bone form in various OTM stages, found in previous works^16,27^, demonstrate that a thorough understanding of bone remodeling also in the later stages of orthodontic induced periodontal remodeling is highly relevant. This offers an important step for the investigations of interrelation between modifications in alveolar bone microstructure and periodontal apparatus.

The strong variability in tooth translational movement and rotation as well as in their directions, found in this study, points out the simultaneous adaptation of force distribution after each small movement during the 5 weeks of OTM. Although the possible small discrepancies in the orthodontic force direction due to a difficult placing of the NiTi coil in mouse model may also contribute to the OTM discrepancy, these cannot fully explain the large differences in the tooth movement. We also point out the possible other factors, such as biological and habitual differences between the animals, i.e. that may influence the progression of OTM. The fact that the estimated rotational movements were found in both directions despite no external changes on NiTi coil setup, indicate that the center of rotation and the nature of movement adapt to the bone changes over the studied time frame and may thus be inconsistent. For these reasons, we propose that periodontal bone remodeling, especially during long-term orthodontic treatment, cannot be simply distinguished by the compression and tension part without an exact estimation of the tooth movement and rotation. Rather a complex remodeling takes place in a wider area around the tooth roots within the periodontal apparatus. A similar estimation may apply also in the case of shorter studies, as a certain degree of tooth rotation cannot be excluded in earlier stages. It should be noted that certain biological aspects may also contribute to the apparent OTM variance in this study, such as possible geometrical differences between both sides of maxillae.

For the evaluation of bone changes following OTM treatment, several studies applied the division to the tension and pressure side method^13–15,20,36^. Still, the tooth movement complexity during a NiTi-coil-induced OTM in small animal research results in high obstacles for the definition of tension and compression sides. Often, the bone resorption mechanism is prevalent on both sides, leading to higher porosity and lowered BMD and Tr.Th in both regions^13,20^. Some studies found the opposite remodeling outcome on both sides, leading to lower porosity and thicker or unchanged trabeculae on the tension side, while thinning of trabecular bone, loss of BV/TV and smaller Tr. Sep appeared in the compression region^14,15,36^. It was usually not examined whether the tooth movement was consistent or whether the constant force led to only translational movement in one direction or whether an additional complete root system rotation took place in the mechanical force application time frame. The OTM mechanism is known to be highly complex and a combination of movements (*i.e.*, translation and rotation) may happen simultaneously and interchangeably^16,33^. Based on an in vivo study of OTM in male Wistar rats up to 31 days, Zong et al*.*^16^ suggested that the tension and pressure side may not be placed on the opposite sides of the tooth root, but rather be adjacent and inter-connected due to the inconsistent OTM rate over the timeline of orthodontic treatment. For that reason, a simple establishing of such areas found in literature and solely based on the the NiTi-coil direction may fall into misleading results regarding various bone remodeling under the opposite mechanical stimuli.

Most of the OTM studies in rodents focus on the alveolar bone in the region of the 1^st^ molar since this is typically the experimental treated molar. Because of the highest expected bone changes in the region between the mesiobuccal and distobuccal root of M1, often a cubic or variably shaped VOI is chosen in this area for the morphometry analysis^18,25,35–40^. In a few studies, the VOI was set up in the region further from the roots^14^ or directly around the roots^21^. Still, the exact position, size and form of VOI vary strongly in the literature. Since studies on differences between the bone remodeling process in various regions of alveolar bone and PDL are missing, there is an open question whether only the alveolar socket of M1 is the most affected bone and whether other processes follow in the surrounding regions. The relatively wide VOI might reduce the statistical power because alveolar bone not affected by the movement might be included too. Some studies suggest applying a thin VOI within the region where bone remodeling is expected to take place (of ~ 100 µm thickness)^21^. It is, however, questionable whether the significant bone changes are restricted only to such relatively small regions close to the stress initiation points or whether wider regions may also be affected, especially after a prolonged orthodontic treatment. Too small VOI may limit the accurate estimation of the parameters, such as Tr.Th and Tr.Sep, if the VOI dimension is comparable to these parameters^13^. On the other side, Zong et al*.*^16^ evaluated the complete alveolar and basal bone around and under M1 and M2. Though, the inclusion of the rather stagnating basal bone into the VOI hinders the detection of smaller changes in the analyzed tissue.

To accurately estimate the change within the alveolar and periodontal bone, we chose to analyze the complete alveolar bone region between the mesiobuccal and distobuccal root (VOI:M1) or between the distobuccal root of M1 and the mesiobuccal root of M2 (VOI:M1-2) and reaching from the furcation up to the tip of the shortest root of M1. In such a case, a maximal VOI is chosen where possible strong changes can be expected while excluding the basal or cortical bone. Also, our VOI dimensions were larger in comparison to the obtained Tr.Th and Tr. Sep. Most importantly, a precise 3D registration ensures an almost identical position of the studied VOI in-between the samples. This allows for a more precise estimation of the morphology parameters as well as for the BMD. To avoid the influence of the cortical bone, the outer boundaries of VOI were set close to the roots center in order to exclude such regions.

The bone resorption as well as bone deposition processes are known to relate to an increased porosity and lower bone volume in the actively regrowing region^41^. Although no modifications in BV/TV after a short OTM (up to 3 weeks) could be detected in some studies in mice or rats^18,42,43^, the most of them showed similar reduction of BV/TV and thus an increase of bone porosity in the OTM group^13,21,24–26,38,44^. In our study, both regions of interest showed a significantly lowered BV/TV indicating a still regenerating phase of the alveolar bone. Due to lacking long-term OTM investigations in mice or rats in literature, this study proves in a novel way that previously observed bone remodeling also proceeds after a relatively long force application. Higher and often increased porosity leads to a stronger intratrabecular connectivity. We could see such an increase in both VOI regions while the connectivity increase was larger in VOI:M1. The higher porosity and connectivity of the OTM affected regions indicate a formation of woven bone along the collagenous PDL fibers in the widened PDL-bone attachment region^32,41^. This initial bone form is known to have an increased porosity comparing to natural alveolar bone and is therefore more susceptible to resorption if inflammation is initiated. In the later stages of bone remodeling, the woven bone will reshape to lamellar bone^45^.

Alongside these findings, the mineral density seems to be mostly reduced after OTM too^13,23,38^, which was also confirmed in a study on orthodontic patients^22^. Interestingly, Wang et al*.*^27^ observed a strong reduction of BMD after only 2 weeks of OTM followed by a turnover and its correction within the next 2 weeks of orthodontic treatment. This phenomenon may explain the findings from other studies in mice or rats, where BMD was not significantly different after 3 or more weeks of OTM^16,18,43^. The possible mineral density regeneration over the period of 5 weeks OTM also corroborates with our findings in both VOIs (i.e., VOI:M1 and VOI:M1-2), where BMD reduction was relatively small or non-significant. The fact that alveolar bone becomes more porous after OTM while mineral density remains relatively stable, indicates the concomitant bone formation and resorption caused by sterile inflammation^16^. That is an opposite regulation to the known phenomenon of pathological processes during osteoporosis^46^ or periodontitis^47^ where both parameters are reduced simultaneously. Thus, the sole BMD parameter is probably not sufficient to study bone changes under mechanical stimulus.

In parallel with bone loss or growth, a thinning of trabeculae has been detected in many studies^16,18,20,43^. Such reduction of Tr.Th obtained in this study after 5 weeks of OTM in both VOIs was greater than found by Holland et al*.*^48^ after 3 weeks of OTM in wild-type mice. Interestingly, this reduction was stronger in the M1 region than in the VOI between M1 and M2. The lowering of Tr.Th is expected, assuming that both bone loss and bone regeneration are happening in VOI:M1, while VOI:M1-2 may prevalently cover a region of bone growth and follows similar trend in other parameters, such as BV/TV, BMD and connectivity. Lower Tr.Th has been found mainly in the compression regions where bone loss process dominates^14,36^. While Dorchin et al*.*^20^ found trabeculae thickening in both tension and compression sides, a constant^36^, decreased^13^, or even increased^14^ value of Tr.Th has been observed on the tension side.

Together with the thinning of trabeculae, a simultaneous increase of Tr. Sep is in agreement with other studies^14,18,36^ and was found to be significant only in VOI:M1 although both areas presented the same trend. Trabecular separation seems to be a less sensitive parameter of bone remodeling and was found stable even after 3 or 4 weeks of OTM in rats^16,43^. Bone regrowth on the tension side resulted in a constant Tr.Sep^36^ or reduction^14^ leading to structures with smaller pores. Our observation indicates that rather a bone loss mechanism is prevalent in both studied regions.

For a more complete view about the changes in periodontium during orthodontic stimuli, this study emphasizes the importance in the precise evaluation of the periodontal ligament. The PDL assumes a regulation function for the force transfer as a bone-PDL-tooth joint-like system^49^. An optimal PDL-space biomechanically permits the effective stress redistribution from the tooth to the adjacent tissue. Despite the fact that the exact role of the PDL tissue in bone remodeling under mechanical load hasn’t been fully understood yet, there is a consensus about the high importance of PDL tissue in such processes as well as about its function as stress absorber and redistributor to the adjacent alveolar bone^41^. Mechanical response and remodeling factors inside the PDL region are expected to be strongly influenced by the heterogeneity of the PDL fiber density and vitality, and thus by the variability of the stress-distribution microregions^50^. The previous analyses of the PDL system indicate the complexity of this collagenous system and its reactions to external stimuli^51^. Still, the mechanism of the PDL-space response to variable magnitudes, directions and durations of loading needs to be examined in more detail. Therefore, the understanding of PDL tissue remodeling as a result of force application is an important progress factor for the orthodontic treatment strategy.

Often, bone morphology and PDL shape are studied only qualitatively and only on selected 2D images missing the complete information from the whole bone and PDL volume^12,14,21,25,29,30,38,43^. Still, for an accurate comparison, the exact position and orientation of the analyzed 2D sections in the control and in OTM treated side is crucial. A volumetric registration of the studied periodontium region shall be an important pre-requisite for such comparative evaluations of PDL thickness. Manually oriented data hinders the validity of the results through the influence of the sectioning angle as well as the position along the molar roots on the estimated PDL thickness. Additionally, the coordinates in which the PDL thickness is measured in 2D are often based on the assumed vector of the orthodontic force applied on the molar tooth. However, our data, confirmed by previous findings^31,33^, demonstrate the complexity of tooth movement during long term OTM and the simultaneous variability of the force direction during the bone and PDL remodeling process. Therefore, a simple orthodontic force definition without an exact estimation of tooth movement and stress distribution may fall into misleading results.

Moreover, the changes in PDL form found in literature are mostly in agreement with our results of PDL thickening due to orthodontic stimuli. An increase in PDL thickness was also detected by Li et al.^43^ in rats only after 21 days of OTM. Significant PDL thickening on the compression side of the distobuccal root of M1 was also observed in mice after 12 days of OTM^25^. A thickened PDL was also found in 1st molar of C57BL/6 wild type mice after occlusal trauma^30^. A strong widening of PDL in both pressure and tension side after 4 and 2 weeks of OTM, respectively, was shown in rats on histology by Hundson et al*.*^38^ and Dorchin et al*.*^20^. Nevertheless, only a qualitative evaluation was performed in the mentioned study. Dynamic changes in the PDL form were observed by Laura et al*.*^29^. Here, the PDL showed a thickening tendency on the tension side and a thinning on the opposite compression side in the first 3 days of treatment. Though, the original PDL thickness was recovered within the next 2 weeks. Such findings go along with the compressed PDL on the compression side and extended PDL on the tension side observed by Shalish et al*.*^12^ after only 4 days of mechanical force application. Both findings indicate the asymmetric changes in PDL thickness caused mainly by tooth movement in the second stage of OTM and assuming no or only negligible effect of rotational forces. Unfortunately, neither work provides verification whether such PDL deformation proceeded also in other root sections.

A full volumetric evaluation of PDL is provided only in a few studies on OTM in mice^32–34^. Here, the PDL thickness was determined by an algorithm that defines the shortest distance from each pixel of the root surface to the surrounding bone surface. For a correct results interpretation, it is important to note that without virtual ‘closing’ of the PDL-bone contact surface, such algorithm also defines the distorted areas and ‘broken pores’ in the alveolar bone as a wider PDL region. Still, the widening of the PDL thickness found in our study, is in agreement with the observations in these studies^32,33^. A volumetric approach was also applied in the study by Wolf et al*.*^21^, where the effect of PDL region extension after mechanical stimuli was confirmed and demonstrated by the significantly lower bone volume inside the 100-µm thick VOI around the molar roots in mice after 11 days of OTM. We note that the additional cementum loss and tooth resorption effect may also contribute to the higher estimated PDL volume after orthodontic treatment^2,21^.

Considerably a similar trend in the changes of PDL thickness and volume found in our study indicate that the PDL deformation on the side with more compression force is not compensated by its extension in the tension force areas after such long application of orthodontic stimuli. Rather the whole PDL system is deformed and remodeled. Our findings imply that the observed changes within the PDL are not solely due to the dislocation of tooth in the alveolar socket and they do not correlate with the tension and compression side after such long mechanical load. Still, more studies are needed to answer the resulting questions, such as what happens with the PDL and alveolar bone reformation after the orthodontic force is removed; how is the alveolar bone and PDL remodeling influenced by different forces; or how is the length and the process of bone and PDL recovery to its original form at different applied loadings. This break-through study was conceived to show the first evidence about an extended time span-remodeling which was successfully reached through clear significant differences in several morphological parameters even in a relatively small sampling. Future studies with higher sample numbers and analysis of periodontal ligament and microstructures of alveolar bone after removing the appliances may be essential for the better understanding of this phenomena and its application in clinical research.

## Conclusion

In this study, we provide a precise evaluation of alveolar bone and PDL-space remodeling after a uniquely long orthodontic treatment of 5 weeks in mice. Parallel bone remodeling trends were found in two regions adjacent to the orthodontically treated molar tooth, indicating a similar process independent on the direction of the original orthodontic load. Such conclusion may also be taken from the extended PDL-space after this long OTM treatment. For a deeper understanding of the bone and PDL remodeling process during the OTM treatment, we propose to estimate the dimensional factors of tooth movement prior to an extended volumetric tissue morphology evaluation. Beside the precise and reproducible definition of the studied alveolar bone or PDL area, the comprehensive analysis of periodontal ligament in 3D is essential.

## Data Availability

The data that support the findings of this study are available from the corresponding author, R.B.C., upon reasonable request.
